# Factors Influencing Adherence to Imatinib in Indian Chronic Myeloid Leukemia Patients: A Cross-Sectional Study

**DOI:** 10.4084/MJHID.2015.013

**Published:** 2015-02-20

**Authors:** Jyotsna Kapoor, Narendra Agrawal, Rayaz Ahmed, Sanjeev Kumar Sharma, Anshul Gupta, Dinesh Bhurani

**Affiliations:** 1 Masters in Clinical Research, Department of Hematology, Rajiv Gandhi Cancer Institute and Research Centre, Sector - 5, Rohini, Delhi, India. PIN 110085; 2 DM, Consultant Hematology, Department of Hematology, Rajiv Gandhi Cancer Institute and Research Centre, Sector - 5, Rohini, Delhi, India. PIN 110085; 3 DM, Consultant Hematology, Hemato-Oncology and BMT Unit, BLK Superspeciality Hospital, Rajendra Place, New Delhi, India, PIN 110008

## Abstract

Adherence to imatinib(IM) is of utmost importance in patients with chronic myeloid leukemia (CML) to maximise treatment effectiveness. The main objective is to measure adherence to IM by evaluating individual patient characteristics, personal behaviour and, treatment related psychological factors influencing adherence behaviour. Hundred patients receiving IM were analysed for adherence behaviour using 9 item Morisky Medication Adherence Scale (9-MMAS). Various factors were assessed for their impact on adherence behaviour. These factors were age, gender, duration of treatment, frequency and dosing of treatment, use of tobacco and alcohol, educational qualification, employment status, monthly income, side effects, financial assistance in treatment, social support, knowledge about medicine and disease, concomitant drug burden, polypharmacy, physician patient interaction, patient educational sessions and prevalence of depression. Seventy five percent of patients were found to be adherent. On univariate analysis, prevalence of depression (p<0.000001), moderate severe depression (p<0.000001), concomitant drug burden (p=0.036) and monthly income (p=0.015) were found to be significantly influencing adherence. The final multivariate model retained prevalence of depression with OR= 10.367 (95% CI, 3.112–34.538) as independent predictor of adherence to therapy. This study suggests that identification and treatment of depression among CML patients may further enhance adherence to IM therapy.

## Introduction

More than a decade ago, revolution came in the treatment of CML with the introduction of the Imatinib Mesylate (IM), a BCR- ABL tyrosine kinase inhibitor. After 5 years of follow – up, continuous treatment of chronic – phase CML with imatinib, as initial therapy, was found to induce durable responses in a high proportion of patients.^1^,^2^ With IM being so effective, the allogenic stem cell transplantation no longer remains the first line treatment, despite being a curative treatment. Though IM is the first line treatment, few drawbacks are associated with its use as it is still not considered to be a curative therapy; it requires indefinite treatment on daily basis and ensuring optimal adherence to treatment for long term. Adherence to medication has been recently defined by an international panel of experts as ‘the process by which patients take their medications as prescribed’ and this process has three main components: initiation, implementation, and discontinuation.^3^

Various studies and several case reports have shown that non adherence to IM is common^4^–^10^,^13^ and intertwined with non-achievement of molecular responses^4^,^5^,^7^,^10^ and event free survival^8^ emphasising strict adherence to the prescribed dose of IM holds paramount importance to maximise treatment effectiveness in CML patients. For example, a Belgian study found that one third of the patients were non adherent and only 14% were adherent to all the prescribed dose. On average, patients with suboptimal response had significantly higher mean percentages of IM not taken (23.2%, standard deviation [SD] 23.8) than did those with optimal response (7.3%, SD 19.3, *P.*005),^5^ Marin et al found that 26% of the patients had adherence rate <90% (considered to be nonadherent) and adherence is a critical factor for achieving molecular responses in patients with CML who achieve complete cytogenetic responses on IM.^4^ Darkow et al found 31% of nonadherence rate among US CML population using electronic data of dispensation of IM and also found non- adherence led to increased healthcare costs.^6^ Adherence to IM have been also studied in the past in Indian population using records of Glivec International Patient Assistance Program (GIPAP) retrospectively in which one third of the patients were found to be non-adherent to IM and concluded that non- adherence to IM adversely affects event free survival (EFS) in chronic phase CML (CP-CML) patients.^8^

There is scarce availability of literature citing the potential reasons for non-adherence to oral anticancer treatment^11^ and few existing data on reasons why CML patients might be non- adherent to IM. Treatment related aspects (side effects, knowledge of disease or treatment, financial cost of treatment etc.), individual patient characteristics (gender, age) and personal factors (social support) have been found to be influencing adherence in chronic illnesses.^11^–^13^ We hypothesized that these factors might affect adherence to IM in CML patients too. Ganesan et al tried to explore reasons of non- adherence to IM in Indian CML patients and assessed age, sex, economic status and Sokal score.^8^ No study has completely investigated the treatment related, individual patient characteristics, personal and psychological factors influencing adherence in Indian patients with CML so far. Therefore, we conducted this personal interview based study to assess the adherence of CML patients using 9 MMAS and to evaluate personal, treatment related, and psychological factor associated with adherence at Rajiv Gandhi Cancer Institute and Research Centre, India.

## Methods

### Study Design and Setting

This study was carried out at Rajiv Gandhi Cancer Institute and Research Centre, Delhi, India. All CML patients over 18 years of age and below 80 years, with ongoing IM therapy for minimum duration of three months, and who visited the outpatient department during a period of February 2013 and May 2013 were considered for inclusion in the study. Patients who were dumb and/or deaf or undergone allogenic hematopoietic stem cell transplant were excluded from the study. The questionnaires were available in hindi and english, the patients who did not understand these languages were excluded. The patients included in the study were taking IM either 400mg/day or 600mg/day or 800mg/day. The patients who were taking 600mg/day or 800mg/day were advised to take half the dose after heavy meal in the morning and the other half dose after heavy meal in the evening to manage the gastric side effects. Optimal sample size was calculated and found to be 84 in accordance with the previous adherence study conducted on Indian population by Ganesan et al (30% of non-adherence rate was found), we approximated the sample size to be 100.^8^ The total number of patients visiting the OPD within this period were 139 and 82.7% (115 patients) of these fulfilled the inclusion criteria.

The questionnaire was translated by official translators in Hindi allowing the majority of patients to undergo personal interview in their native language. The patients were given oral and written information regarding the study when asked to participate. After giving oral and written consent for participation, the study coordinator personally interviewed the patients using questionnaires in their preferred language. This study was approved by the Institutional Review Board of our centre. This study was conducted in accordance with latest version of Declaration of Helsinki.

### Questionnaires

The questionnaire used consisted of 9-MMAS (to measure adherence behaviour), *additional questionnaire* (to assess the factors influencing adherence except depression) and PHQ-9 ( to assess prevalence of depression). The questionnaire asked about adherence behaviour, socio-demographic background, knowledge about disease and medicine, social support, physician patient relationship, role of patient educational sessions, side effects of medicine, financial assistance in treatment, concomitant drug burden, polypharmacy, details about therapy, and depression. *Additional questionnaire* was partly devised from questionnaire, previously used by Jonsonn et al^9^ and questions regarding role of patient educational sessions, polypharmacy, financial assistance in treatment and concomitant drug burden were added in view of our cohort. The internal consistency reliability of the combined questionnaire to assess the factors influencing adherence (additional questionnaire and PHQ-9), using Crohnbach α was found to be 0.72.

### Adherence Behaviour

The 9-item Morisky Medication Adherence Scale (9-MMAS), a standardised test, was used to measure adherence, with scores ranging from 1–13, where 13 indicates perfects adherence. This test has been developed from the well validated Morisky Green Test and the eight item MMAS.^15^,^16^ The internal consistency reliability of the English version of 9- item MMAS, measured by the Crohnbach α, had a value of 0.89.^15^ The 9- item MMAS is composed of 9 questions explores adherence behaviour based on forgetfulness, negligence, interruption of drug intake and restart of drug intake when symptoms worsen. Patients scoring 11 or above in the summary score were classified as adherent. This definition of adherence is based on how patients theoretically would have completed the MMAS if they had taken at least 95% of prescribed doses.

### Factors Influencing Adherence

Socio-demographic background composed of 8 questions asking about gender, age, marital status, employment status, educational qualification, monthly income, and use of tobacco or alcohol in any form. For example, with regard to employment status, a question was asked ‘Do you work?’ with an option of ‘Yes/No’. Knowledge about Medicine and Disease composed of 5 questions along with subparts to find out whether the respondents have basic knowledge about their disease and treatment. For each correct answer ‘1’ was scored. Support given by family, friends and colleagues was assessed using 10 questions comprising of Yes/No option. A healthy and regular physician patient interaction was assessed using a set of 7 questions followed by a Yes/No option except one question. Questions included were ‘Do you visit your physician at regular intervals?’, ‘Do you feel the physician is very helpful to you?’ ‘Do you trust your physician?’ etc. Patients were interviewed whether they have attended the last patient educational session on CML and if yes, did they found it helpful to find out the role of patient educational sessions on adherence. Patients were questioned about being financially assisted in treatment, if so, and then what were the means of assistance. Concomitant drug burden was defined as the assumption of additional drugs related to diseases other than CML may affect the adherence to IM (Yes/No). Polypharmacy was defined as taking at least one alternative medicine apart from IM for CML (Yes/No) may affect the adherence to IM. Commonly used alternative medicines were from ayurvedic, homeopathic and unani system of medicine. Patients were also questioned about the side effects if they ever had with the use of imatinib and if they had, the side effects were recorded accordingly. The prevalence of depression among CML patients was evaluated with a Patient Health Questionnaire-9 (PHQ -9), a validated and standardized instrument with good specificity and sensitivity. The PHQ-9 focuses on the nine signs and symptoms of depression from DSM-IV. In addition, the sum score of PHQ-9 (0–27) is used for screening purposes and for measuring depression severity. As a severity measure the PHQ-9 score can range from 0–27, since each of the 9 items can be scored from 0 (Not at all) to 3 (Nearly every day).

## Statistical Analysis

The quantitative variables were presented with mean and SD, however the categorical variables in frequencies along with respective percentages. The reliability of all the domains of the questionnaire was tested by Cronbach alpha. When comparing adherent with non-adherent patients, in the univariate analysis, chi-squared test was used to analyze categorical data (gender, use of tobacco/smoking, use of alcohol, employment status, educational qualifications, patient educational sessions, financial assistance in treatment, Side effects of Imatinib, prevalence of depression, concomitant drug burden, polypharmacy, dose of imatinib and frequency of dosing of imatinib), the independent t-test was used to compare means (age, knowledge about medicine and disease, social support, physician patient interaction and duration of prescription of imatinib) and Mann-Whitney U test was used to compare Monthly income.

Multiple logistic regression analysis was used to identify factors associated with adherence. For variable selection in the model, the backward stepwise likelihood ratio method was used to perform regression analysis with probability less than 0.3. Data were analyzed using SPSS version 21.0 (2012, IBM Corp, Armonk, NY, USA) and p value <0.05 was considered of statistical significance.

## Results

**Figure 1:**
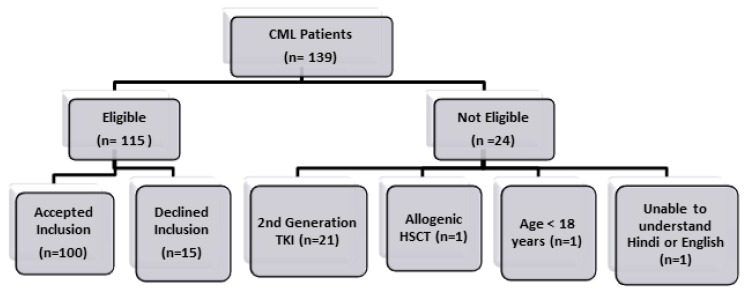
Patient Recruitment Details

In this study, 100 out of 115 eligible patients completed the interview (response rate 86.9%) (Figure 1). 51% of the respondents were interviewed in Hindi language.

### Descriptive Statistics

Descriptive statistical data of 100 patients analyzed are present in Table 1. The majority of the respondents were male (63%) and the mean age was 41.08 years (range 18–70) and median duration of imatinib therapy was 30 months (range 3–101).

### Adherence Behaviour

All patients included in the study (n=100) completed the 9-MMAS. The median Morisky Score of 100 patients included was 12 (Range; 7–13). 75 (50 male and 25 female) out of 100 patients had Morisky score ≥ 11, therefore classified as adherent. Twenty two percent of the respondents scored 13, i.e. perfect adherence. Forty six percent of the respondents had special routine or reminder system which helps them taking medication. Ninety three percent patients took their medicine prior to the day of interview. None of the patients had summary score <5. Four out of twenty five non adherent patients had summary score between 5 and 8.

### Comparison of variables with Adherence

The univariate analysis is presented in Table 2 and 3. Among the quantitative variables, monthly income of the patients was found to be significantly associated with adherence (p-value 0.015). Among the categorical variables, prevalence of depression (p value <0.000001), moderate severe depression(p<0.000001) and concomitant drug burden (p value = 0.036) were found to be significantly associated with adherence behaviour. Non depressed people were more likely to be adherent (84.4% vs 43.5%). Patients with no concomitant drug burden were more likely to be adherent (78.8% vs 53.3%).

The results of the logistic regression analysis of factors associated with adherence (9-MMAS summary score ≥ 11), adjusted for covariates are presented in Table 4. The variables included in the study were age, knowledge about medicine and treatment, physician patient interaction, those who attended patient educational sessions, male, depressed patients, smokers, alcoholics, educational qualifications, employed patients, patients who had side effects, being financially assisted in treatment, had concomitant drug burden, having polypharmacy and dosage of imatinib. Full data were available for all the 100 patients, who were included in the logistic regression analysis. Prevalence of depression among CML patients remained independently associated with adherence (OR= 10.367, 95% CI 3.112–34.538).

## Discussion

The objectives of the study were to assess the prevalence of adherence to imatinib treatment in Indian CML patients, to evaluate the factors associated with adherence. In this sample, 75% of the respondents were classified as adherent. Factors associated with high adherence were no concomitant drug burden, no prevalence of depression and monthly income. As the questionnaire was also available in Hindi, participation of patients who could not understand English was encouraged. The response rate of patients was found to be fairly high (86.9%).

Optimal adherence to imatinib therapy is crucial to maximize treatment effectiveness,^4^,^5^,^7^,^8^ however the ability of the physician to recognize adherence is poor.^19^ Given the scanty data of CML literature, we selected the possible factors to be associated with the adherence behaviour based on previous studies in other chronic medical illnesses.^12^,^18^,^19^,^23^ The percentage of patients found to be non-adherent in our study (i.e. 25%), seems consistent with previous data indicating non adherence rates of 25 to 50%.^19^ Also, it is difficult to make the comparisons regarding prevalence of non adherence in other studies as this fluctuates as a function of methods used. However, our study support previous findings that adherence to imatinib therapy is far from optimal (i.e 75 % of patients have adherence rates ≥ 95%) in CML patients.^5^ As per our knowledge, only one study in a small cohort of 38 patients have found ‘good’ adherence to imatinib therapy.^9^

Negative significant association between the adherence and the prevalence of depression among the Indian CML cohort was observed with a p value <0.00001. 23% (n=23) of patients were found to be depressed, out of which none of the patient was severely depressed. 47.82 %(n=11), 34.78% (n=8) and 17.4% (n=4) patients were found to be mildly, moderately and moderately severely depressed. We further analyzed the severity (mild, moderate and moderately severe depression) of depression with adherence and found moderate severely depressed patients to be significantly associated with non-adherence (p<0.000001). Our study revealed that non depressed patients are more likely to be adherent (84.4% vs 43.5%). Prevalence of depression was found to be the only factor to be associated with adherence through multivariate logistic regression analysis with odds ratio of 10.367 with 95% confidence interval of odds ratio to be between 3.112 and 34.538. Given the paucity of data in the CML literature regarding the negative association between adherence and depression, our findings are thus consistent with the meta-analysis performed by Di Matteo et al which included 12 articles about depression and noncompliance to medical treatment and 13 articles about anxiety and noncompliance to medical treatment revealed a significant and substantial relationship between depression and non-adherence to medical treatment prescribed for chronic illnesses.^22^ A recent meta analysis on the depression and medication adherence of patients with chronic diseases in U.S population by Grenard et al estimated the odds of a depressed patient being non-adherent are 1.76 times the odds of a non-depressed patient across 31 studies and 18,245 participants.^24^

In our cohort, concomitant drug burden was found to be negatively associated with adherence to imatinib therapy (p value − 0.036). Out of 15 patients on concomitant drugs, only 8 patients (53%) were found to be adherent. Though our results contrasts with the results obtained worldwide, which states that concomitant drug burden has a positive association with adherence to imatinib therapy in CML patients.^5^,^13^ Noens et al showed an association between more medication to be taken daily and better adherence to imatinib therapy.^5^ A qualitative study by Eliasson et al 23 reported that adherent patients referred to taking imatinib as being part of their daily routine, possible to speculate that patients who are already taking medications for other diseases might be facilitating in fitting CML therapy into their regular overall medication taking schedule. However, we might have observed such a contrasting result because the concomitant drug burden in the previous studies was fairly high (41.16% in Efficace. F et al)^13^ unlike our study (15%) and only 46% of the patients in the 9-MMAS reported that they had a special routine or a reminder system to facilitate their medication taking behaviour.

58% patients were found to be working in our cohort of Indian CML patients with a mean monthly income of Rs.20,912.93 (range Rs.550–2,00,000). Our results showed monthly income to be associated with adherence to imatinib therapy (p value- 0.015) through univariate analysis but this was found to be insignificant when logistic regression analysis was performed.

There is conflicting evidence in the literature whether age influences adherence in CML patients. A study of 87 patients by Marin et al,^4^ showed that younger patients have lower adherence rate whereas older patients with a median age of 53.8 years had a adherence rate of greater than or equal to 90%. Unlike our study, did not show that increasing age positively influences adherence (p value − 0.795).

A study of Darkow et al^6^ on 267 patients showed adherence to be influenced by gender, non adherence was significantly higher in women; in the present study this difference was not observed (p = 0.234). Santoleri et al concluded that frequency of dosing does not influence adherence to drug therapy.^20^ Though the imatinib is once a day dose, but patients prescribed 600mg/day or 800mg/day of imatinib were advised to take half the dose in morning and other half in evening to manage the gastric side effects. Similar results were obtained through this study (p value − 0.536). Imatinib therapy is prolonged and previous research has shown that adherence for long – term drug therapies are lower, often no more than 40–50 %,^13^ but our study reflected no significant association between adherence and duration of prescription (months) of imatinib( p= 0.743). The side effects of imatinib are relatively mild, dyspepsia (21%) and edema (21%) was found to affect the CML patients the most. As these side effects are mild, adherence was found not to be influenced by side effects (p=0.051). Richardson et al showed that patient educational programs including information on disease and expected side effects were associated with better survival in patients with hematologic malignancies.^26^ Moon et al reported that a counselling programme was effective in improving compliance in CML patients receiving imatinib.^27^ But, our study did not reflected the similar results, as we found patient educational sessions did not play a significant role in influencing adherence (p value- 0.325)

Backward step wise multiple logistic regression analysis was used to find the independent predictors of adherence. Initially, all the independent variables were included in the model. Further, non-associated variables were dropped one by one step wise and finally age, knowledge about medicine and disease, physician patient interaction, patient educational sessions, prevalence of depression, financial assistance and concomitant drug burden were selected at 10^th^ step with probability less than 30%. The criteria of 30% was based on the assumption to find the closely related variables with adherence. Among all the selected variables, only depression was significantly (OR 10.367; 95% C.I, 3.112–34.538) associated with the adherence. However, other independent variables showed the closeness to the adherence. Marin et al showed that younger patients have lower adherence.^4^ In HIV patients, the perceived very good contact with health care was found to be associated with adherence to antiretroviral treatment.^14^ Efficace et al found concomitant drug burden as an independent predictor of adherence in CML patients to IM.^13^ Moon et al reported that a counselling programme was effective in improving compliance in CML patients receiving imatinib.^27^

This paper has number of strengths including, selection bias is likely to be limited as the proportion of non-respondents was fairly small (15 of 115). A response rate of almost 87% is fairly good and the proportion of eligible patients was also high (115 of 139). No internal attrition was found. For appropriate results, the sample size approximation was priorly done in accordance with the adherence study conducted on Indian population.^8^

This paper, however, also has potential limitations. First, we might have missed additional patient related and psychological factors that might have found to be related to adherence in patients with other diseases.^25^ Second, we used non validated questionnaire to assess the factors influencing adherence except depression and third, it is possible that additional measures of adherence could have further contributed to a more sensitive definition of adherence in our study. However, the methods available for measuring adherence all have different strengths and weaknesses; because of the complexity of the adherence behaviour and problems with bias, none is optimal and self-report methods provide a good estimation of medication adherence in an inexpensive manner over a possible breadth of distribution and also have great advantages over other methods.

These potential limitations notwithstanding, we are confident our results extend findings of previous research in the field of adherence and investigation of factors influencing adherence in CML on IM to suggest key potential determinant of adherence behaviour. Physicians are encouraged to pay attention to factors identified in this study could help to promptly identify patients who might be at a heightened risk of non adherence.

## Figures and Tables

**Table 1:** Socio Demographic, Clinical, Personal, and Treatment Related Factors

Variable	N(%)/Mean±SD	Variable	N(%)/Mean±SD	Variable	N(%)/Mean±SD	N	Median (Range)
Gender -Male	63	Female	37	__	__	100	__
Age, at entry (years)	41.08±12.33	__	__	__	__	100	40(18–70)
Primary Education or less	18	Middle to Higher secondary education	48	Graduation and above	34	100	__
Use of Tobacco	5	No use of tobacco	95		__	100	__
Use Of Alcohol	9	No use of Alcohol	91	__	__	100	__
Married	83	Single	10	Other	7	100	__
Employed	58	Unemployed	42	__	__	100	__
Never Had Side Effects	51	Had Side effects	49	__	__	100	__
Financial Assistance In treatment	96	No Financial Assistance In Treatment	4	__	__	100	__
Concomitant Drug Burden	15	No Concomitant Drug Burden	85	__	__	100	__
Depression	23	No depression	77	__	__	100	__
At entry, Chronic CML	97	Accelerated CML	3	Blast CML	0	100	__
400mg/day IM	81	600mg/day IM	13	800mg/da y IM	6	100	__
Monthly Income (Rs./month)	20,912.93±311 15.95	__	__	__	__	58	13,500 (550– 2,00,000)
PHQ-9 Score	3.65±4.32	__	__	__	__	100	2(0–19)
Duration Of IM(months)	34.14±23.39	__	__	__	__	100	30(3–101)
Adherent	75	Non Adherent	25	__	__	100	__
Knowledge about Medicine and Disease Score	7.65±1.87	__	__	__	__	100	8 (3–12)
Social support Score	6.15±1.71	__	__	__	__	100	6(2–10)
Physician Patient Interaction Score	7.74±0.90	__	__	__	__	100	8(3–9)
Polypharmacy	2	No Polypharmacy	98	__	__	100	__
Patient educational Sessions (Attended)	32	Not Attended	68	__	__	100	__

**Table 2:** Comparison of quantitative data with adherence

Background variables	Adherence group	Non adherence group	t value	P value
	*Mean±SD*	*Mean±SD*		
Age (years)	40.89±12.10	41.64±13.21	0.261	0.795
Knowledge about Medicine and Disease	7.56±2.02	7.92±1.32	0.832	0.408
Social Support	6.3±1.65	5.68±1.84	1.596	0.114
Physician Patient Interaction	7.78±0.77	7.60±1.22	0.891	0.375
PHQ-9 Score	2.48±2.88	7.16±5.84	5.283	0.000
Duration Of Prescription (months)	34.58±23.43	32.80±23.70	0.329	0.743

**Table 3:** Comparison of Categorical Variables with Adherence

Variables		Adherence	Category	Total	p Value
		No adherence N(%)	Adherent N(%)		
Sex	Female	12(32.4)	25(67.6)	37	**0.234**
	Male	13(20.6)	50 (79.4)	63	
Use of Tobacco/Smoke	No	23 (24.2)	72(75.8)	95	**0.429**
	Yes	2(40)	3(60)	5	
Use Of Alcohol	No	22(24.2)	69(75.8)	91	**0.547**
	Yes	3(33.34)	6(66.67)	9	
Educational Qualification	Upto Primary	5(27.8)	13(72.2)	18	**1**
	Middle to Higher secondary	11(22.9)	37(77.1)	48	
	Graduation and above	9(26.5)	25(73.5)	34	
Employment Status	No	14(33.34)	28(66.67)	42	**0.103**
	Yes	11(18.96)	47(81.03)	58	
Patient educational sessions	Not Attended	19(27.94)	49(72.05)	68	**0.325**
	Attended	6(18.75)	26(81.25)	32	
Side effects	No	8(16.32)	41(83.67)	49	**0.051**
	Yes	17(33.34)	34(66.67)	51	
Financial assistance	No	2(50)	2(50)	4	**0.241**
	Yes	23(23.95)	73(76.04)	96	
Concomitant Drug Burden	No	18(21.17)	67(78.82)	85	**0.036**
	Yes	7(46.67)	8(53.33)	15	
Polypharmacy	No	1(50)	1(50)	2	**0.412**
	Yes	24(24.49)	74(75.51)	98	
Depression	No	12(15.58)	65(84.41)	77	**<0.000001**
	Yes	13(56.52)	10(43.47)	23	
Dosage of IM (mg/day)	400mg/day	19(23.45)	62(76.54)	81	**0.468**
	600mg/day	4(30.76)	9(69.23)	13	
	800 mg/day	2(33.34)	4(66.67)	6	
Frequency of dosing	Once a Day	19(23.45)	62(76.54)	81	**0.464**
	Twice a Day	6(31.57)	13(68.42)	19	
Mild Depression	No	20(22.47)	69(77.52)	89	**0.98**
	Yes	5(45.45)	6(54.54)	11	
Moderate Depression	No	21(22.82)	71(77.17)	92	**0.90**
	Yes	4(50)	4(50)	8	
Moderately Severe Depression	No	21(21.87)	75(78.12)	96	**<0.000001**
	Yes	4(100)	0	4	

**Table 4:** Multiple logistic regression analysis to identify the predictors of adherence

Predictors^a^	AOR	95% CI
Age	1.041	0.989–1.096
Knowledge about Medicine and Disease	0.782	0.566–1.082
Physician Patient Interaction	1.458	0.763–2.788
Attended Patient Educational Sessions^b^	0.504	0.140–1.811
Depressed Patients^b^	10.367**	3.112–34.538
Financially Assisted Patients^b^	6.451	0.727–57.240
Presence of Concomitant Drug Burden^b^	3.813*	0.843–17.254

** p <0.001.

AOR = adjusted odds ratio. CI = confidence interval.

* p<0.05.

a - variable is continuous in nature.

b - the variable was collapsed into dichotomous variable with the options of Yes/No
